# Detection of inadvertent passage of guide wire into the false lumen during thoracic endovascular aortic repair of Type B aortic dissection by transesophageal echocardiography

**DOI:** 10.1186/s40981-022-00539-y

**Published:** 2022-07-16

**Authors:** Mohamed Eissa, Asadollah Mir-Ghassemi, Sudhir Nagpal, Hesham F. Talab

**Affiliations:** 1 Department of Anesthesiology and Pain Medicine, The Ottawa Hospital, 501 Smyth Rd, Ottawa, ON K1H 8L6 Canada; 2 Department of Anesthesia and Intensive Care, Ain Shams University, Cairo, Egypt; 3 Department of Vascular Surgery, The Ottawa Hospital, Ottawa, ON Canada

**Keywords:** TEE, Vascular, TEVAR, False lumen, Endoleak

## Abstract

**Background:**

Thoracic endovascular aortic repair (TEVAR) has become a widely accepted treatment strategy for patients with thoracic aortic pathologies. We present a case of TEVAR where transesophageal echocardiography (TEE) played a crucial role for adequate placement of an endovascular stent graft.

**Case presentation:**

A 71-year-old male received TEVAR for type B aortic dissection. TEE detected both true/false lumens with an intimal tear. A guidewire was inserted into the descending aorta via the left femoral artery; however, angiography failed to identify the precise location of the tip of the guidewire. TEE detected the guide wire passing through the intimal tear into the false lumen, promoted the surgeon to manipulate and advance it to the true lumen, followed by placement of a stent graft. The patient was hemodynamically stable through the whole procedure.

**Conclusion:**

TEE was crucially important for detecting the precise location of the guidewire and preventing complications during TEVAR.

**Supplementary Information:**

The online version contains supplementary material available at 10.1186/s40981-022-00539-y.

## Background

Transesophageal echocardiography (TEE) has a significant role in the intraprocedural decision making improving the outcomes and decreasing postprocedural complications of thoracic endovascular aortic repair (TEVAR). Its use with fluoroscopy and angiography can reduce the radiation exposure and contrast load. TEE can easily identify the true lumen, the false lumen. and the intimal tears communicating between them. It can confirm the correct position of the stent-graft guidewires within the true lumen before stent deployment [1]. Moreover, after stent deployment, TEE is used to confirm the technical success of TEVAR by the reduction of blood flow into the aneurysmal sac or the false lumen and to exclude the presence of endoleaks and new intimal tears in adjacent aortic segments [1, 2]. We present a case of TEVAR where TEE was helpful in advancing the guide wire into the true lumen of a dissected aorta, followed by placement of the stent graft in the optimal position.

## Case presentation

**Fig. 1:**
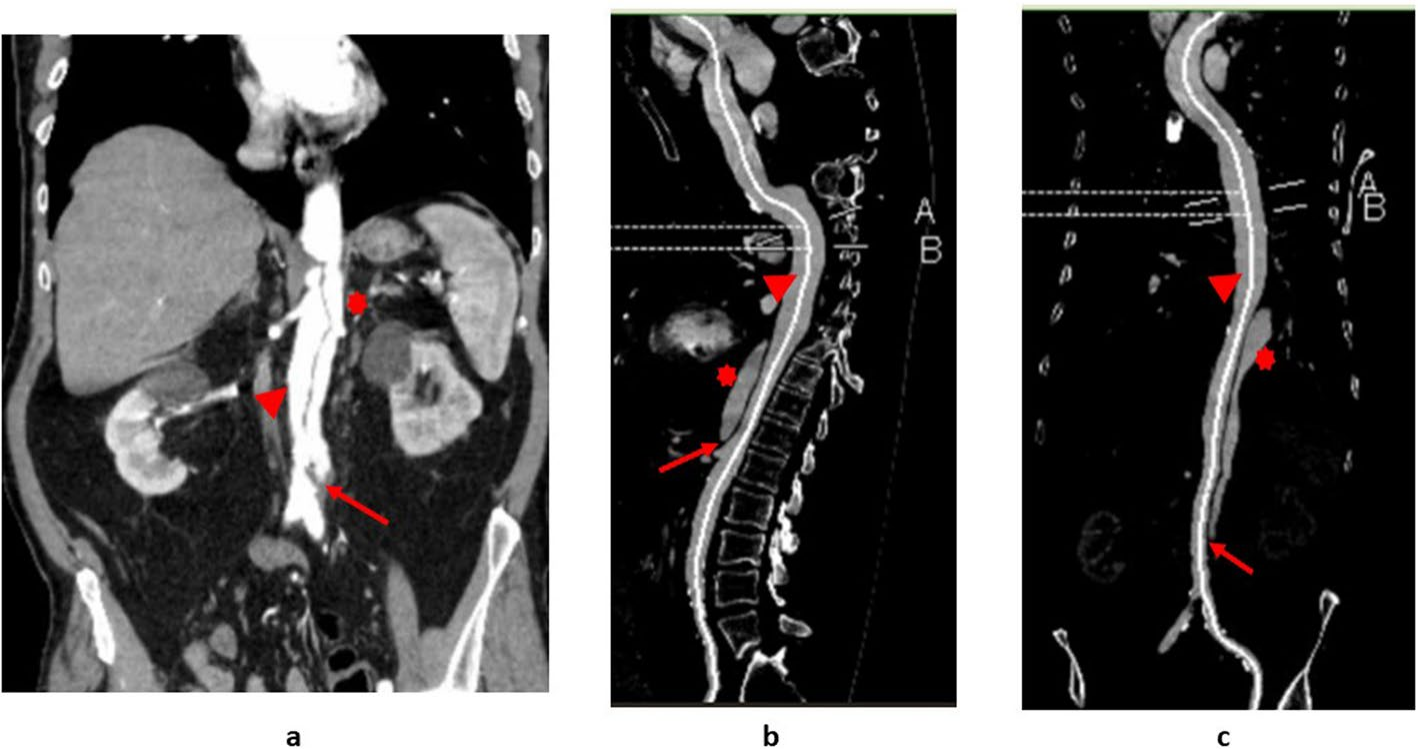
Preoperative CT scan of the chest (**a**), abdomen (**b**) and pelvis (**c**) with IV contrast showing true lumen (red arrowhead) and false lumen (red asterix) as well as the dissection flap (red arrow)

Written informed consent was obtained from the patient to publish this case with accompanying images. A 71-year-old male patient was scheduled for TEVAR for aortic dissection. He was known to be hypertensive with a type B aortic dissection with no neurological deficits and has been receiving conservative therapy. His preoperative CT showed comparable extent of his aortic dissection from just distal to the left subclavian artery origin into the aortic bifurcation, with a stable dissection flap (Fig. 1). However, it showed interval aneurysmal dilatation of the mid descending thoracic aorta to a total diameter of 5.2 cm compared to 3.7 cm over 3 months during his follow-up. This was a rapid degenerative change constitutes a strong relative indication for early endovascular stent graft repair to promote favorable aortic remodeling and prevent further significant aneurysmal dilatation and rupture.

In the operating room, in addition to standard Canadian Society of Anesthesiology monitors, arterial line and depth of anesthesia monitor (Entropy) was applied. Lumbar drain was also inserted at the level of L4–L5 for spinal cord protection. Following induction of general anesthesia, a TEE probe was inserted, and central venous access was achieved with a 6 FR introducer through which a transvenous pacing wire was placed for overdrive pacing to facilitate stent deployment.

**Fig. 2:**
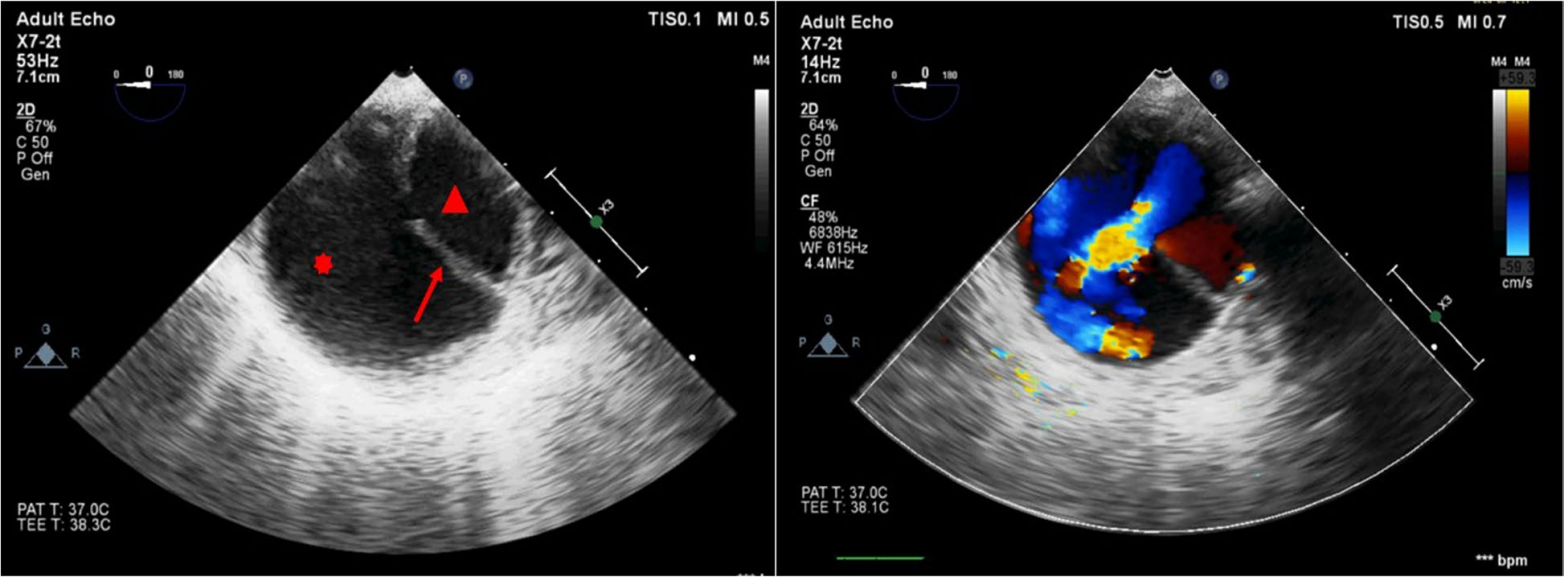
Transesophageal echocardiography of the descending aorta in short axis. True lumen (red arrowhead) and false lumen (red asterix) are separated by an intimal flap of the dissected aorta, with an intimal tear (red arrow) at the level of the mid descending thoracic aorta (left) and color flow mapping confirming blood flow from true lumen to false lumen through the intimal tear (right)

Standard TEE examination was performed, and true and false lumens were identified. At the proximal part of the aneurysm an intramural thrombus was identified in the false lumen. At the level of the mid thoracic descending aorta, an intimal tear was found communicating the true lumen with the false lumen. This was confirmed by using color flow Doppler mapping (Fig. 2). Using real time dynamic fluoroscopy, the surgical team were able to utilize the tip of the TEE probe as a marker to help the identification of the level of this intimal tear.

**Fig. 3:**
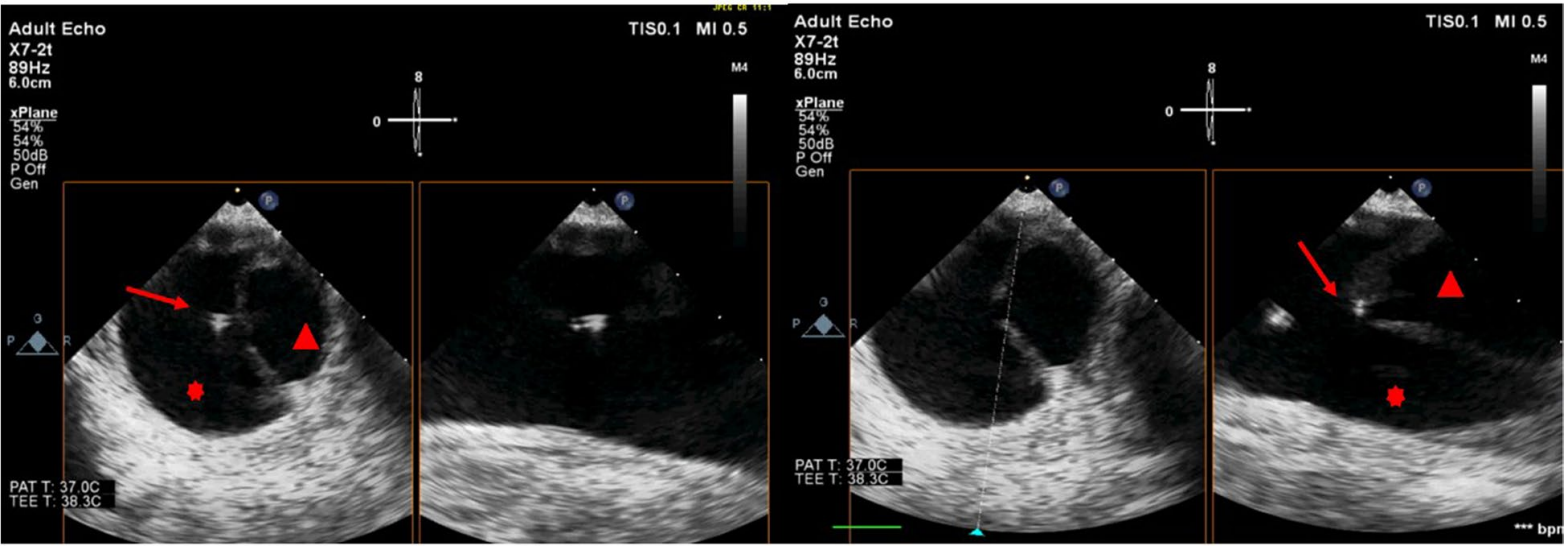
Transesophageal echocardiography of the descending aorta in long and short axes. True lumen (red arrowhead) and false lumen (red asterix) are separated by an intimal flap of the aortic dissection, with a guide wire passing through the intimal tear from the true to the false lumen (red arrow)

Endovascular repair was performed through cannulation of the left femoral artery. Under angiographic guidance, a guide wire was inserted to establish vascular access and facilitate stent graft deployment. TEE identified the wire location within the false lumen while intraoperative angiography failed to recognize this issue at this stage. Moreover, we were able to see the guidewire passing through the intimal tear into the false lumen by TEE. This was confirmed by using the TEE in both long and short axes to exclude artifacts (Fig. 3). Further wire manipulation under TEE guidance led to proper positioning of the guide wire within the true lumen. The surgical procedure continued, and the stent graft was deployed. After stent deployment, TEE examination was repeated to exclude any endoleaks or new dissection in the adjacent aortic segments by both 2D and color flow mapping. The patient was hemodynamically stable through the whole procedure and was successfully extubated at the end. Patient stayed in the ICU for 2 days to be discharged later to the floor and then home with no complications.

## Discussion

It was in the early 1950s when the first surgical repair of an abdominal aortic aneurysm (AAA) involving the visceral vessels was reported [3]. Huge innovations in aortic surgery were then made by DeBakey and Creech [4]. However, despite marked improvements in the surgical techniques and perioperative care, the morbidity and mortality associated with open surgical repair of the thoraco-abdominal aorta remained high. This may be attributed to the need for aggressive surgical exposure, aortic cross clamping with the associated physiological stress and ischemia reperfusion injury of tissues below the cross clamp. Moreover, the risk for spinal cord ischemia, renal dysfunction, respiratory failure, myocardial infarction, and coagulopathy were also high with the open repair [5].

Endovascular repair of AAA was first introduced in 1991. Nowadays, thoracic endovascular aortic repair (TEVAR) has become a widely accepted treatment strategy for patients thoracic aortic pathologies [5]. This minimally invasive procedure has much lower mortality and morbidity than the conventional open surgical repair. This allowed high-risk patients, who would have never been considered as candidates for open repair, to have a therapeutic option.

False lumen endovascular stent placement is a devastating complication of TEVAR. It may lead to true lumen compression with resultant mesenteric, renal, and lower extremity malperfusion as well as extending the dissection, which is often fatal [6]. Rapid intervention and intraoperative detection of such event is paramount to prevent serious complications.

Endovascular stent-graft placement for aortic dissection treatment requires precise knowledge of true and false lumen during guidewire and stent passage. Confirming the accurate placement of guidewires, catheters, and stents are mandatory all through the course from the femoral artery to the aortic arch for a safe procedure. The wires can begin in the true lumen and inadvertently cannulate a fenestration to arrive in the false lumen without operator knowledge.

That is why appropriate anatomical knowledge from the CT scan is required before the operative procedure as understanding visceral vessel reliance on true or false lumens is essential. Knowing which iliac vessel contains true or false lumen, perhaps both, is also mandatory.

**Fig. 4:**
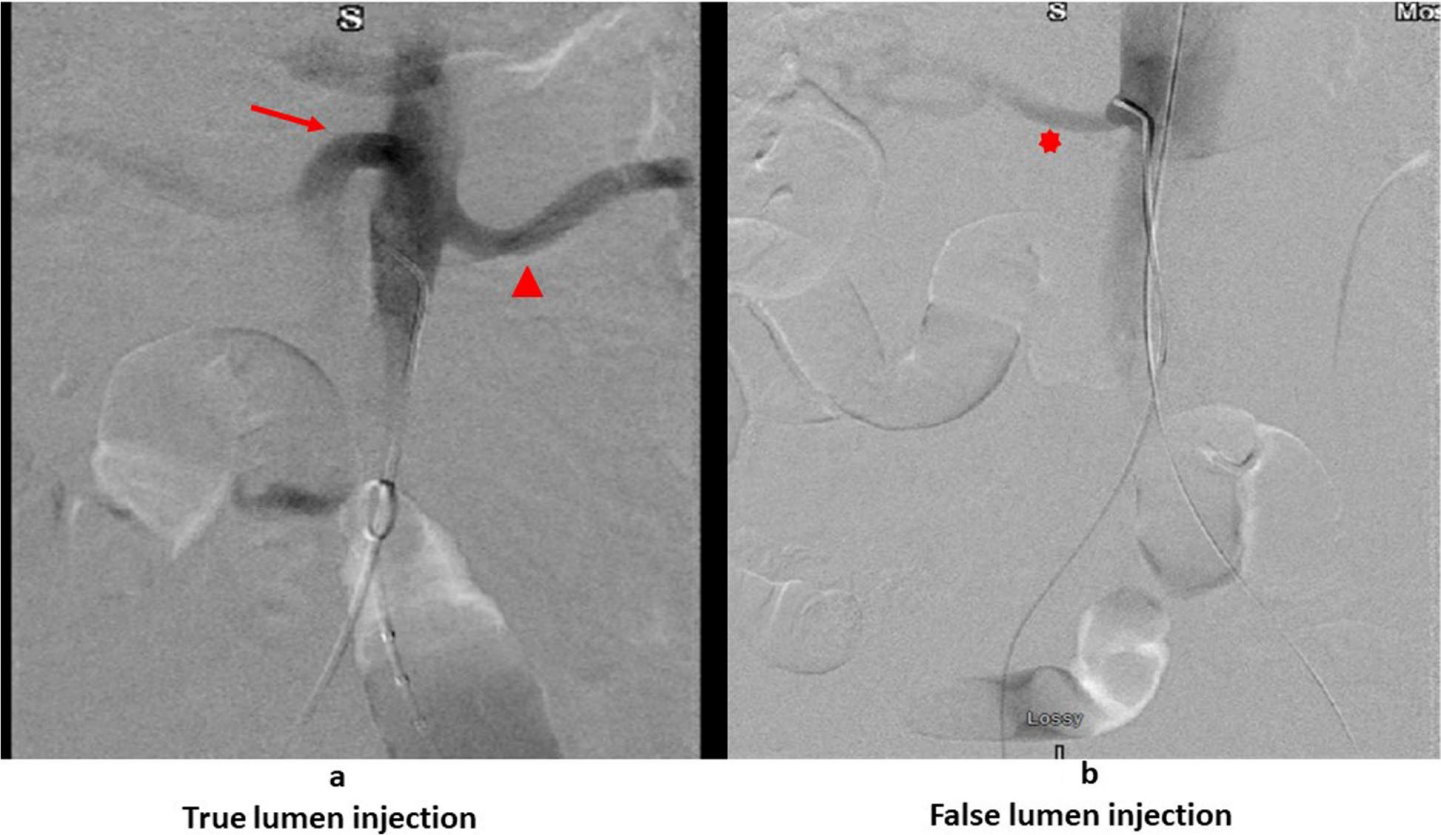
**a** Contrast images of the descending aorta. True lumen injection, showing superior mesenteric artery (red arrow) and left renal artery (red arrowhead). Pigtail now pushed into chest to be followed by Transesophageal echocardiography. **b** Contrast images of the descending aorta. False lumen injection, showing right renal artery (red asterix). Requires catheter withdrawal and cannulation of true lumen

The common femoral artery is entered in a retrograde fashion and the guidewire can cannulate into the false or true lumen. This should be confirmed through retrograde femoral injection angiogram assistance. The wire is then brought to the visceral segment and an angiogram ascertained. Knowing which visceral vessels arise from the true and false lumens is paramount. Once the pigtail catheter visceral artery angiogram is obtained, a visceral artery comparison is made to the CT scan. For example, if the superior mesenteric artery and left renal artery originate from the true lumen and the right renal artery from the false lumen, a true lumen angiogram will immediately visualize the superior mesenteric artery and the left renal artery (Fig. 4a). If the right renal artery is predominantly visualized (Fig. 4b), this indicates a false lumen catheter injection, and then the guidewire is returned to the iliac vessels for cannulation into the true lumen. Once confirmation of the true lumen status is made, the pigtail catheter, not a standalone guidewire, is pushed into the lower thoracic aorta. TEE can then follow the pigtail from there into the proximal arch confirming true lumen passage throughout the thoracic aorta as well. The pigtail catheter is a safer method to traverse the thoracic aorta as an extensive fenestration would be required to cross mistakenly into the false lumen. Regardless, confirmation by TEE or intravascular ultrasound (IVUS) is required. They can easily identify distal fenestrations and visualize the aortic branches. Pigtail catheter angiography together with TEE can be a quick, accurate and inexpensive alternative for IVUS, without requiring the special equipment or expertise needed for IVUS [7].

The true lumen confirmation must be done in the abdomen and chest separately as this patient was having multiple fenestrations in the thoracic and abdominal aorta from the subclavian down to the iliac vessels. Once the pigtail is pushed into the chest and across the arch a stiffer wire is used to deploy the stent-graft. The same method is used to bring up another wire to the aortic arch for appropriate angiograms.

Previous cases of inadvertent deployment of the endovascular graft into the false lumen have been described before that were not detected intraoperatively and led to marked morbidity and mortality of the patients after with mesenteric ischemia, elevated liver function tests, and worsening kidney functions [7, 8].

To our knowledge, this is the first case with TEE images and loops showing the guidewire passing through the intimal tear into the false lumen. We present this case to remind other practitioners of how TEE use in TEVAR can be very crucial by early detection and prevention of such a devastating complication that can really worsen the patient’s outcome.

## Supplementary Information

**Additional file 1: Supplemental video file 1.** Transesophageal echocardiography of the descending aorta in long and short axes. True and false lumens are separated by an intimal flap of the aortic dissection, with an intimal tear at the level of the mid descending thoracic aorta: Guide wire can be seen passing through the intimal tear from the True lumen into the False lumen.

## Data Availability

The data used in this case report are available from the corresponding author on reasonable request.

## Abbreviations

TEVAR: Thoracic endovascular aortic repair
TEE: Transesophageal echocardiography
AAA: Abdominal aortic aneurysm
IVUS: Intravascular ultrasound
